# The Comparative Virulence of the Tubercle Bacillus from Human and Bovine Sources

**Published:** 1902-03

**Authors:** Mazyck P. Ravenel

**Affiliations:** Lecturer on Bacteriology, Veterinary Department, University of Pennsylvania; Bacteriologist of the State Live-Stock Sanitary Board of Pennsylvania


### THE COMPARATIVE VIRULENCE OF THE TUBERCLE BACILLUS FROM HUMAN AND BOVINE SOURCES.[1]

By Mazyck P. Ravenel, M.D.,
LECTURER ON BACTERIOLOGY, VETERINARY DEPARTMENT, UNIVERSITY OF PENNSYLVANIA; BACTERIOLOGIST OF THE STATE LIVE-STOCK SANITARY BOARD OF PENNSYLVANIA.
(*From the Laboratory of the State Live-stock Sanitary Board of Pennsylvania.*)

(Concluded from page 81.)

### Comparative Virulence of Bovine Culture H and Human Culture K for Puppies and Pigs when Introduced through the Alimentary Tract.

*Puppies.* Four puppies, eight weeks old, from the same litter, were selected, two of which received a suspension of milk of bovine culture H and two a similar suspension of human culture K, equal amounts being given on ten days, six days in succession, with an interval of seven days, then four days in succession. The regular food was sterilized milk, bread, and boiled beef.

Only one of the four puppies died, one of the two which received the human culture K, death occurring after fifty-seven days. There was a generalized tuberculosis, most marked in the lungs and liver. The other puppy was killed after eighty-five days. The only lesions found were small cheesy nodules on the surface of one lung, in parotid and mesenteric glands.

The two puppies fed with bovine culture H showed no ill effects whatever, and were killed on the eighty-fifth and eighty-seventh days, respectively. Both were in good condition, the only lesions found being in the parotid and mesenteric glands, which contained minute caseous areas, none larger than 2 mm. in diameter.

*Pigs.* As with the puppies, four pigs from the same litter, eight weeks old, were taken, two being fed with human culture K and two with bovine culture H, equal amounts of a suspension in sterile water being soaked into bread and given for ten days, with an intermission of only one day. The regular food was sterilized milk, ground oats, bran, and corn-meal.

All four of the pigs died, the average life of the two fed with human bacilli being one hundred and eighteen and a half days against one hundred and a half days for the two which received the bovine culture. One pig of each pair lived one hundred and twenty-three days, dying on the same day. The total difference in gain of weight was only half a pound. All showed post-mortem a generalized tuberculosis.

While some differences were noticed in the extent of the disease in various organs, it cannot be said that these were such as to indicate a greater virulence of one culture than the other. In all the mesenteric glands were extensively diseased, while in one only, fed with bovine bacilli, was there ulceration of the mucous membrane of the intestine. Three of the four showed tuberculous ulceration of the tonsils. The remaining one presented no gross changes in this gland, but microscopically areas of necrosis, apparently in the lymph-follicles, were found, and sections stained with carbol-fuchsin revealed large numbers of tubercle bacilli. The two pigs which lived longest, one bovine and one human, both had extensive involvement of the joints, so that for several weeks before death walking was almost impossible.

The result of this experiment is inconclusive as far as the puppies are concerned, except as showing that they are readily infected through the alimentary tract. The pigs showed practically an equal susceptibility to the two cultures. The ease with which they were infected, and the extent of the lesions produced in so short a time, were both most striking features, and should serve to put farmers on their guard against feeding the swine on milk from tuberculous cattle. In Pennsylvania neglect of this precaution has led to considerable loss in several instances.

*Recovered Cultures.* The cultures included in Table III. were all recovered from animals which had been inoculated or fed with cultures H and K. Their virulence was tested only for rabbits and guinea-pigs. The most striking thing to be noticed in the results is the increase in virulence in the human bacillus recovered from the hogs K (D) and K (E). The average length of life for guinea-pigs after inoculation with the original culture K was thirty-eight days, while the rabbits were killed after eight months and twenty-three days, showing successfully resisted infection. On the other hand, the culture recovered from the hogs killed guinea-pigs in twenty-nine and one-half and thirty-two and one-third days, and rabbits in seventy-one and one-half and sixty-two and one-half days, respectively, showing a virulence for those animals which corresponds to that seen in bovine cultures.

Culture H (E) was recovered from a nodule on the hand of Dr. S. H. G., who accidentally inoculated himself while making a post-mortem on one of the goats which had succumbed to the original culture H. Particular interest attaches therefore to this culture. No marked difference can be detected in the virulence of this culture as compared with the original.

Considerable difference has been noted in the readiness with which the recovered cultures grew in the early generations. H (A), H (B), H (D) and H (F) all grew vigorously from the start, the three last growing without any stirring of the tissue on the original tubes, while H (G) grew on tubes sealed with paraffin—a result which I have been able to obtain only with the human bacillus, with this exception. The morphology of all the recovered cultures correspond closely to the original.

### Comparison of Other Human and Bovine Cultures. (Table IV.)

### HISTORY OF CULTURES.

*Culture F* (*Bovine*). Obtained from a large tumor on the diaphragm of a cow which had been slaughtered for beef. The tumor was 13 cm. long and from 7 to 9 cm. in diameter. On the surface were a number of grape-like masses of pearly lustre. On section numerous caseous areas were found, from which an emulsion was made. It contained many tubercle bacilli. A guinea-pig was inoculated intraperitoneally May 16, 1899. Death occurred on June 8th, and from its tissues a second pig was inoculated intraperitoneally. This pig was chloroformed July 31st, and a third pig inoculated in the same manner, which was chloroformed August 21st and cultures made from spleen and omentum. Scanty growth was observed on September 27th. Under the microscope the bacilli were thick, short, and showed little tendency to beading, staining deeply and uniformly. The further history of this culture is given in the paragraph on "Retention of Characteristics in Culture."

*Culture Q* (*Bovine*). Obtained from the lungs of cow No. 5986. This cow was brought to our department on July 6, 1898, for experimental purposes, being considered then an advanced case of tuberculosis. She was emaciated and coughing badly. Many tubercle bacilli were found in the mucus she coughed up, which were long, thin, and much beaded. She died February 12, 1900. The lungs were infiltrated throughout, many of the areas having undergone caseation and calcification. It was difficult to understand how the animal lived as long as it did. The mediastinal glands formed a tumor 35 cm. long by 15 cm. in diameter, which was cheesy on section. The mesenteric glands were enlarged and cheesy. A guinea-pig was inoculated in the peritoneal cavity on November 15, 1899. On November 30th the animal was killed and cultures made. Growth was observed first on January 10th, and sub-cultures made on the same date. Growth was always scanty.

*Culture QQ* (*Bovine*). Was obtained from the mesenteric gland of cow No. 5986. An emulsion from the centre of the gland was inoculated into the peritoneal cavity of a guinea-pig February 13, 1900. On March 5th it was chloroformed and cultures made. Growth was observed on April 12th, and sub-cultures were made on April 17th. Growth was more abundant than that on the preceding culture (Q), but still scanty.

*Culture T* (*Bovine*). Was obtained from milk sent in for examination by a dairyman in Philadelphia. It was inoculated into the peritoneal cavity of a guinea-pig January 24, 1900. The guinea-pig was chloroformed on April 6th. With an emulsion of its spleen two other guinea-pigs were inoculated intraperitoneally on the same date. One of these was chloroformed on April 28th and cultures made. Growth was observed first on June 23d and cultures made the same day. Growth was abundant and luxuriant

*Culture U* (*Human*). Obtained from a child three years of age, whose death was due to tubercular meningitis. Autopsy. Lungs and bronchial lymphatic glands full of miliary tubercles. Anterior mediastinal glands much enlarged. Spleen extensively involved, liver less so. On abdominal surface of diaphragm were a number of flat, yellow nodules. Mesenteric glands enlarged, and contained old, yellow, cheesy nodules. One small tubercle found in right suprarenal. Purulent and cheesy nodules found in meninges and encephalon. The culture was obtained from mesenteric glands.

The pathologist, Dr. Hand, was uncertain as to the origin of this case.

Guinea-pigs were inoculated intraperitoneally on June 15, 1900. One was killed on July 23d, and with its tissues a second was inoculated intraperitoneally. This was chloroformed on September 12th and cultures made. On October 11th, no growth having taken place, the blocks of tissue were transferred to fresh serum and stirred. On December 4th growth was first observed, and on December 15th sub-cultures were made. Growth has been rapid and abundant.

*Culture W* (*Human*). Obtained from a child nine months of age, of scrofulous parentage. The cervical glands on both sides were enlarged. Autopsy. A double bronchopneumonia was found. One small tubercular nodule was found on surface of left lung, several at apex of right lung. Bronchial glands not enlarged.

Tonsils normal in size, but glands on each side of pharynx extending in front of and behind the sternomastoid muscle were enlarged, tuberculous, and several contained creamy pus. Three mesenteric glands were enlarged and cheesy; an ulcer was found in the jejunum and another at the ileocæcal valve. Peyer's patches and the lymphatic glands about cæcum were enlarged. Spleen and liver contained tubercles. The mesenteric glands were employed for the isolation of the culture.

This case was considered by Dr. Hand as being probably of tonsillar or pharyngeal origin.

Guinea-pigs were inoculated intraperitoneally on October 5, 1900. From one which died on January, 1901, a second was inoculated intraperitoneally. This was killed March 4th and cultures made. Growth was first observed on April 12th and sub-cultures made the following day.

*Culture BB* (*Human*). Obtained from a child seventeen months of age. Death was due to tubercular meningitis. Autopsy. Lungs normal, except posterior and part of right upper lobe, which was consolidated, red on section, and had excess of connective tissue. Bronchial and mediastinal glands not enlarged. Spleen, liver, and kidneys show pearly tubercles. Two feet from the lower end of the ileum is an ulcer 1 by 2 cm., the long diameter transverse to axis of gut. Nodules are seen on peritoneal surface. Four small ulcers, two above and two below, are found in the ileum. Mesenteric glands enlarged, cheesy, and some purulent. Meninges show yellow tubercles most marked along longitudinal fissure, in choroid plexus, and over cerebellum. The mesenteric glands were used in obtaining the culture.

This case is considered by Dr. Hand the clearest one of primary intestinal tuberculosis ever seen by him.

Guinea-pigs were inoculated intraperitoneally with an emulsion made from the mesenteric glands on March 9, 1901. One was chloroformed on April 19th and cultures made. Scanty growth on one tube was found in June and sub-cultures made.

The results given in Table IV. show for all the bovine cultures a greater virulence than for any of the human cultures. The average life of the guinea-pigs inoculated with human bacilli was more than twice that of those inoculated with bovine. All the rabbits which received human cultures gained in weight and had finally to be killed, while for the bovine they died and presented post-mortem extensive lesions, the lungs and kidneys being the chief seats of the disease in all animals.

### PART II. TESTING THE PATHOGENIC POWER OF TUBERCULOUS MATERIAL OF HUMAN AND BOVINE ORIGIN.

In addition to the study of a number of pure cultures obtained from man and cattle, we have examined the pathogenic power of tuberculous material from the two sources also, making a series of inoculations which are as nearly parallel as possible. The results of these tests are shown in Tables V. and VI. The plan was to inoculate a series of animals with tuberculous tissues rubbed into a smooth suspension, and at the same time to inoculate a number of guinea-pigs with the same, which on their death would furnish material for another series of inoculations parallel with those made directly from the human and bovine tissues. It was believed that the material from guinea-pigs would more nearly represent pure cultures of the respective organisms than would be obtained from the tuberculous organs of man and cattle, and that the two sets of animals would lend themselves to an interesting comparison, besides fulfilling the primary object of comparing the virulence of human and bovine material.

The plan was carried out for the bovine material, but failed for the human, as all the guinea-pigs died within forty-eight hours after inoculation. The second series of animals were inoculated with human material from another source and obtained at a later date. The results, while not entirely comparable, are nevertheless interesting and valuable.

*Objections to Use of Tuberculous Material.* There are several disadvantages in using tuberculous material which were not overlooked in planning this work. 1. It is impossible to give accurate doses, for although our suspensions may be made of an equal opacity, and counts of equal amounts show approximately the same number of bacilli, there is no way of determining what proportion of these bacilli are capable of multiplication. 2. Material from man almost always contains other bacteria. Our knowledge of mixed infections is too scant to enable us to estimate the part played by those other species in determining the result; whether they inhibit or aid the action of the tubercle bacillus. The same is true of bovine material, although to a less extent, the tuberculous foci being more apt to remain inclosed. On the other hand, infection under natural conditions is always through such mixed material, and this method of inoculation approaches in some degree the conditions we meet in practice.

*Source of Bovine Tuberculous Material.* The bovine tuberculous material was obtained from a cow six years old, which had shown symptoms of tuberculosis for a long time, and was finally condemned and killed. She showed, post-mortem, a generalized involvement of the thoracic cavity with some invasion of the abdominal organs. In the lungs but little tissue approaching normal was found, and the pleuræ were studded with small nodules. Many of the tuberculous areas in the lungs had undergone caseation. The mediastinal glands were enormously enlarged and caseous. Scattered over the peritoneum were many nodules. The liver was considerably involved, and the portal and mesenteric glands were enlarged and caseous. Other abdominal organs showed no macroscopical lesions. Nodules were taken from the pericardium, mediastinal, and mesenteric glands, lung, and pleura. After thorough trituration in a mortar, each part separately, they were mixed, sterile water added, and the whole strained through cheese-cloth. With this material a series of animals was inoculated, and at the same time a number of guinea-pigs. These all contracted tuberculosis, four dying on the fifteenth day. Portions of their organs were prepared in the same way and used for the inoculation of a second series of animals, as seen in Table V.

*Sources of Human Tuberculous Material.* This material was obtained from three bodies, the lungs only being used. Two of the three were cases of well-marked acute miliary tuberculosis, the other a case of chronic phthisis, all adults. The material was prepared for injection in the same way as described for the bovine, the suspension being made as nearly of the same thickness as possible. The first series of animals was inoculated with the tissues from one of the cases of acute miliary tuberculosis on August 27th. A number of guinea-pigs were also inoculated, with the object of having their tissues for a second series of inoculations, as with the bovine material, but they all died of septicæmia within forty-eight hours, preventing the execution of this plan. With the exception of the pigs, and the calves inoculated with sputum, the other animals were inoculated from the two cases mentioned above.

*Method of Inoculation.* In all the animals the injection was made into the lung, the field of puncture being shaven, washed with soap and water, next with alcohol and ether, and lastly with a 1 : 1000 solution of bichloride of mercury. The site of puncture was, for the horses, between the eighth and ninth ribs; for the pigs, sheep, and dogs, between the sixth and seventh ribs; and for the cats, between the fifth and sixth ribs.

### Condensed Post-mortem Notes.

*Horses. Bovine Material. Two Animals.* One died after fifty-four days, with loss of 195 pounds ; the other was killed after sixty-six days, having lost sixty pounds. In both the disease was mainly in the thoracic cavity. In the one inoculated with material direct from the cow, both lungs were involved throughout. In the other the tuberculous process was confined to the lung into which injection was made, and was marked only near the site of the inoculation.

*Horses. Human Material. Two Animals.* Both had to be killed. One was entirely normal, not even the point of inoculation being discernible. In the other the tuberculous process was confined to an area 5 cm. in diameter, with the point of inoculation as a centre.

*Pigs. Bovine Material. Four Animals.* All died, with an average length of life of fifty and three-quarter days. The two which received the bovine material direct lived sixty-eight and one-half days as against thirty-three days for those inoculated from the guinea-pigs. In all the lungs were the chief seat of the disease, all showing an acute miliary tuberculosis of both organs. In the two inoculated directly from the cow the abdominal cavity was invaded to a limited extent, seen only in the mesenteric glands.

*Pigs. Human Material. Six Animals.* Five died and one was killed. The average length of life of the two inoculated directly from man was eighty-eight and one-half days ; for the three inoculated with tissues of other pigs which died the average was twenty-two days. In the first two the lungs were invaded throughout with miliary tubercles, and one showed areas of pneumonic character. In neither was the abdominal cavity involved. Of the four pigs inoculated with material from the other pigs which had succumbed to inoculation with human tissues, three presented a diffuse tubercular pneumonia, rapidly fatal, one living only thirteen days. The remaining animal was normal with the exception of the right lung and pleura. Both visceral and parietal pleuræ were largely covered with nodules 2 mm. to 12 mm. in diameter, many of them caseous. The lung was dense throughout and contained many caseous areas, most marked near the surface.

*Sheep. Bovine Material. Two Animals.* Both died, the one inoculated directly with bovine material in forty-four days, the other in thirty-two days. Both were considerably emaciated, and in both the disease was confined almost entirely to the thoracic cavity, the spleen being the only abdominal organ involved, and this only slightly so. In both the lungs showed hepatization, more marked in the one inoculated with the tissues of the guinea-pig.

*Sheep. Human Material. Two Animals.* Both were killed 202 days after inoculation. One was entirely normal, with the exception of an area in the right lung 5 cm. in diameter at the point of inoculation, in the centre of which was a caseous abscess, the pus containing many tubercle bacilli. The second animal was normal throughout.

*Dogs. Bovine Material. Two Animals.* The dog which received the bovine material direct became markedly ill, much emaciated, and died after 184 days. The pleural surfaces of both lungs were covered with minute nodules, and were almost completely consolidated. In the right lung at point of inoculation was a small abscess, 25 mm. in diameter.

The dog inoculated with tissues of guinea-pig showed little effect from it, and was killed after 245 days. The only lesion found was an abscess cavity 12 mm. in diameter at point of inoculation in right lung, scrapings from the wall of which contained many tubercle bacilli.

*Dogs. Human Material. Two Animals.* One killed after 229 days. The only evidence of disease was the presence of numerous minute nodules on the pleural surfaces of both lungs and a few on the pericardium. The lungs were normal otherwise.

The second dog began to cough after six weeks, lost flesh rapidly, and died on the sixty-third day. Both pleuræ contained six ounces of sanguinolent pus. The lungs were the seat of an extensive miliary tuberculosis, and at point of inoculation in right lung was an abscess cavity 7 cm. long by 2.5 mm. in diameter. The abdominal organs were not involved.

*Cats. Bovine Material. Two Animals.* Both became rapidly ill, one dying in twenty and the other in thirty-one days. In both the lungs were the seat of an acute miliary tuberculosis. In the one which received the bovine material direct the spleen contained a number of cheesy areas. In the other the mesenteric glands were enlarged and cheesy. The more rapid progress of the disease in the latter was probably due to its smaller size and the larger dose.

*Cats. Human Material. Two Animals.* One cat died after two days and has not therefore been included in the averages. The second was killed after 193 days and found to be normal throughout. Not even the point of inoculation could be determined.

*Summary.* Twelve animals were inoculated with bovine material. Ten of these died, while two survived—the horse and the dog which received the tissues of the guinea-pig.

Fourteen animals were inoculated with the human material, of which eight died and six were killed.

For most of the animals the tuberculous material direct from the cow seemed to be the more virulent. The sheep and pigs were exceptions to this, the pigs notably so, those receiving the tissues of guinea-pigs dying in thirty-three days, as against sixty-eight and one-half days for the others. The averages of life after inoculation given in the table, as well as the greater mortality, show strikingly the increased potency of the bovine material over that from man.

This difference is further brought out by the extent and character of the lesions produced by the one and the other, as shown in the post-mortem notes and in Table VI.

*Infection of Calves with Human Tuberculous Sputum.* The results of this experiment are included in Table V., the details, which are of considerable interest, being supplied here.

Calf No. 5984, age five weeks, weight 108 pounds, was inoculated intraperitoneally with 10 c.c. of sputum from an advanced case of tuberculosis at the University Hospital, on July 29, 1898. The patient from whom the sputum was obtained was a young adult who had been sick about a year and a half, and had been expectorating freely for one year. A cavity had been observed in the left lung about eight months before the sputum was obtained. Death occurred soon after. Beyond some slight elevation of temperature this calf showed no effect whatever from the inoculation. On November 3, 1898, it was tested with tuberculin, but gave no reaction. When it was killed on January 14, 1899, it weighed 258 pounds, an increase of 150 pounds. It was in good condition and showed no tuberculous lesions in any part of the body. The site of inoculation could not be determined.

Calf No. 8499, age five weeks, weight 131 pounds, was inoculated on July 29, 1898, with 10 c.c. of sputum of a patient at the University Hospital. This patient was an adult male—a miner by occupation. He had been under treatment for nine months. At this period the apices of both lungs were consolidated. He had a cough for two years, lost flesh, and had one severe hemorrhage. He expectorated a large amount of mucopurulent sputum.

This calf showed an elevation of temperature which continued almost without intermission for three months, reaching as high as 105.8° F. in August and September. On November 3, 1898, it was tested with tuberculin and gave a reaction. It was killed on January 9, 1899, weighing 265 pounds, an increase in weight of 134 pounds. It was in fair condition. There was a nodule in the peritoneum at the site of inoculation. In the omentum were numerous calcareous nodules the size of a hazel-nut, and one softened nodule the size of a hen's egg, which contained an inspissated purulent material, and five smaller ones of the same character. The lungs, liver, and spleen were normal, but on the pleural surface of the diaphragm there was a deposit of fibrin which contained several well-defined nodules. On the abdominal surface of the diaphragm was a small amount of grape formation.

Calf No. 8049, age about four weeks, was inoculated on May 16, 1898, intraperitoneally with 10 c.c. of sputum from an advanced case of pulmonary tuberculosis at the University Hospital. The sputum contained many bacilli. Beyond a slight cough, which was noticed in July, it showed no symptoms of discomfort and grew rapidly. It was tested with tuberculin on July 30th and gave good reaction. It was killed on August 1st, weight 340 pounds, condition good; large amount of fat. On the surface of both lungs and on the pleural surface of the diaphragm was a fibrinous deposit. In the cervical lobe of the right lung a few small nodules were found on section. The mediastinal and bronchial glands were somewhat enlarged. The omentum contained a few nodules, and one about one inch in diameter, which had undergone softening, the contents being rich in tubercle bacilli. The spleen showed a number of nodules. About one-half of the mesenteric glands were enlarged and showed cheesy degeneration. An emulsion from the centre of the softened nodule in the omentum and from portions of the spleen were made and 20 c.c. injected intraperitoneally into calf No. 9843, four weeks old, weight 108 pounds, on August 2d. Beyond a slight cough, noticed on October 10th, the animal showed no ill effects from inoculation. It was killed on January 10, 1899; condition good. The omentum was adherent to the peritoneum at the point of inoculation, and contained some ten nodules about the size of a pea. In every other respect the animal was entirely normal.

Calf No. 8050, age four weeks, was inoculated on May 1, 1898, with sputum from an early case of pulmonary tuberculosis at the University Hospital. The sputum contained a large number of tubercle bacilli. Soon after inoculation the temperature of the calf rose and continued high, with some remissions, until it was killed. Its appearance was bad, the coat dry and rough, the respiration rapid. It was tested with tuberculin, but the temperature was too high for results. It was killed on August 1st, weighing 190 pounds. On the surface of both lungs there was a slight deposit of fibrin, and on section a number of hemorrhagic areas were observed in both. The mediastinal and bronchial glands were enlarged and congested. The abdominal cavity contained about twelve ounces of bloody serum. The peritoneum was thickly studded over its entire surface with nodules from 1 mm. to 12 mm. in diameter, fibrous in character. In many places these nodules had massed together, forming tumors, some 5 cm. in diameter, which were dense and fibrous. The spleen contained many nodules, both on the surface and throughout its substance. The whole omentum was thickly studded with nodules from 2 mm. to 12 mm. in diameter, and besides which there were three large masses, dense and fibrous in character, two of which were 15 cm. long by 7 cm. wide, and 12 mm. thick ; and the third 7 cm. long by 6 cm. wide, by 4 cm. thick. The abdominal surface of the diaphragm was thickly studded with nodules, fibrous in character. The mesentery was thickened and contained many nodules of small size. The appearance was that of a typical case of grape or pearl disease. The mesenteric and mediastinal glands were enlarged and somewhat caseous. Twenty c.c. of an emulsion made from these glands, which contained a large number of tubercle bacilli, were injected on August 2d into the peritoneal cavity of calf No. 9846, four weeks old, weight 132 pounds. The animal showed no ill effects whatever from inoculation and was killed on January 10, 1899. A careful post-mortem examination showed it to be normal in all respects.

*Summary.* Four calves of nearly the same age received intraperitoneally 10 c.c. of human tuberculous sputum from different sources, but in all cases containing a large number of tubercle bacilli.

One showed no ill effects from the injection except a slight rise of temperature, and when killed the autopsy was entirely negative.

Of the other three, two had persistent high temperature following the injection, but only one showed marked illness otherwise. Post-mortem examination proved that all had become infected with tuberculosis, the lesions in two being quite extensive. From each of these two a second calf was inoculated intraperitoneally with an emulsion made from well-developed nodules. In both cases the emulsion was rich in tubercle bacilli, and a large dose (20 c.c.) was injected. The result was absolutely negative in one animal and practically so in the other.

Since both calves received a much larger number of tubercle bacilli in the emulsion than those injected with the sputum, we are led to conclude that the result in the latter was due to a mixed infection which operated to the advantage of the tubercle bacillus.

The attempt to infect calves with human sputum by the digestive tract failed wholly. Two young calves (Table V., Nos. 8074 and 8096) were given from 30 to 60 c.c. of sputum containing many tubercle bacilli on eleven days. Some disturbance of digestion resulted, but when killed no trace of tuberculosis was detected.[1]

*Indications for Further Investigation.* In the further elucidation of this question, cultures should be isolated from as many cases of primary intestinal tuberculosis as possible, and especially those cases in which there is reason to suspect infection by meat or milk. Having obtained the cultures, it may well be asked whether or not we are in a position to determine positively the origin of the offending organisms. Are the differences which have been noted in culture, morphology, and virulence sufficiently marked and persistent to make differentiation possible? This I doubt. In my judgment much more work must be done before we shall be able to determine the origin of a given culture of the tubercle bacillus by examination of it in cultures. At present we know practically nothing of the influence of the human body on the tubercle bacillus, nor what changes may be induced in its morphology, cultural peculiarities and virulence, by residence in the human tissues, nor what length of time is necessary to induce such changes, if induced at all. In my studies I have, as shown in Table III., recovered the tubercle bacillus, both human and bovine form, horses, dogs, swine, goats, and in one instance had the rare if not unique opportunity of recovering the bovine organism from man after accidental inoculation. These recovered cultures have all been carefully compared with the originals, and while some differences have been observed and noted in the remarks following the table, with possibly one exception, they have not been of a marked or distinguishing character. In the case of the man the bacillus remained in his tissues from January 1st to February 27th, fifty-eight days. The recovered culture was

[1] This experiment was conducted by Dr. W. G. Shaw, to whom all the credit is due.

practically identical with the original. This is, however, a shorter time by a great deal than elapses between intestinal infection in children through food and their death, and does not enable us to draw conclusions as to these cases. Like the culture recovered from animals, it indicates that the tubercle bacillus is quite tenacious of its characteristics, as a rule. We have noted constantly that the bacilli found in scrapings from the various organs of the animals inoculated with bovine cultures have been long and beaded, though the culture used was of the short and unbeaded type. The recovered cultures, however, on blood-serum have always resembled the original. In a series of examinations of material coughed up by cows I failed always to find bacilli of what has been described as the bovine type. In fact, the longest tubercle bacilli I have ever observed, except in old cultures, were seen in some specimens of this material. The virulence of this material was greater considerably than human sputum ordinarily is, though the comparison was not made with accuracy.

*Retention of Characteristics in Culture.* On blood-serum with 5 per cent. of glycerin the tubercle bacillus from bovine sources will, as a rule, retain its morphological and cultural characteristics for a long time. Culture H has now been grown for two years (July 6, 1901), and is of the sixteenth generation. Beyond an increased vegetation, no marked change can be detected in it. With a single exception (Culture F) the same may be said of all the cultures isolated. This culture was isolated with some difficulty, and so scant was the growth for six generations that I was several times on the point of abandoning it. Only in the sixth generation was growth enough obtained for experimental inoculation. Considerably more growth took place in the seventh, and the eighth grew luxuriantly, since which all sub-cultures have been abundant. From the eighth generation on serum cultures were made on 5 per cent. glycerin-agar, and an abundant growth obtained on this medium in the first transfer.

Coincident with this increase of vegetative power came a marked change in the morphology. From being short, thick, and staining evenly, it is now long, more slender than in the early growths, and shows marked beading. In other words, from being a typical "bovine" culture, it has during the past ten months so changed that it can now pass as a typical "human" culture. It will be observed, by reference to Table IV., that its pathogenic power for guinea-pigs and rabbits was not as great as is usually found in bovine cultures.

*Culture in Collodion Sacs.* The method of culture in the body of living animals, which has been productive of such brilliant results in the hands of the French, offers much assistance in the solution of the problem before us. It has enabled Nocard[4] to demonstrate the possibility of so modifying the mammalian tubercle bacillus that it becomes like the avian organism in culture and pathogenicity. We are now attempting to modify two feebly virulent human cultures by residence in the peritoneal cavity of cattle.

Culture M was kept for seven months in the abdominal cavity of a heifer, inclosed in collodion sacs, two sacs being used. On removing the sacs but little multiplication of the bacilli was noted. Cultures were recovered directly from the sacs. In the first transfer the cultures grew more rapidly and abundantly than the original culture, but the morphology of the individual bacilli cannot be said to have changed to a noticeable degree. The virulence was not increased. Rabbits were not killed by subcutaneous inoculation. Guinea-pigs died after an average of forty-two and one-third days. A calf inoculated intraperitoneally showed no ill effects and gave no reaction to tuberculin after seven weeks.

Culture Nasua Narica (Coati) of Theobald Smith, inclosed in collodion sacs, was kept in the peritoneal cavity of a yearling heifer for eleven months. On removal there was considerable increase in the growth, especially in one sac. Cultures were recovered from the sacs, and showed some marked changes, especially in manner of growth. On both blood-serum and glycerin-agar the growth was most rapid, at least twice as rapid as usual for this culture. On serum it covered the surface in seven to ten days, as a thick, white, moist layer, almost cottony near the water at bottom. On glycerin-agar the growth was more dry and wrinkled, but very rapid. The bacilli stain more evenly, and are on the whole shorter, though long forms are seen. Guinea-pigs and rabbits inoculated subcutaneously are still living after four months. A calf which received 4 c.c. of a milky suspension in the jugular vein reacted to tuberculin after ten weeks.

The experiment is being continued, the culture recovered from the capsule having been placed again in the peritoneal cavity of another calf.

No conclusions can be drawn as yet, though the indications are that both cultures have become less strictly parasitic rather than more so by the procedure.

*Interpretation of Results.* Accepting it as proven that the bovine tubercle bacillus has, as a rule, considerably greater pathogenic power than the human bacillus for a large majority of experimental animals, How should we interpret this in regard to man? Is it fair to conclude that this increase of virulence will hold good for man also? Until the contrary is proven, or until good reason for believing the contrary is shown, it is in my judgment right that this conclusion be held, at least as a working hypothesis. I am aware of the objections to this view that will be raised by some, and acknowledge freely that it cannot be accepted as conclusive. Virulence is, no doubt, a factor which is relative to the subject, and exaltation of virulence for one species does not necessarily prove an increased virulence for other species. Indeed, the reverse is true in some instances.* However, it cannot be denied, as a general rule, that when the virulence of a pathogenic organism is increased for one animal it is increased for all that are naturally susceptible to its action.

The tubercle bacillus is unique in the extent of its pathogenic activity, both by direct experimental inoculation as well as by infection under what may be considered more or less natural conditions. The list of animals in which tuberculosis has been observed in parks and zoölogical gardens is appalling, the discoveries of Dubard[5] and others showing that not even the cold-blooded animals are exempt from this universal scourge. While it may be said of the tubercle bacillus that in cultures in the laboratory it is unusually tenacious of its characteristics, it is certain that in nature it has a wide range of adaptability as a pathogenic agent. Hence for the tubercle bacillus, perhaps more than for any other known microbe, we are justified in believing that an exaltation of virulence for practically all experimental animals will hold good in the case of man also.

The question can be determined definitely only by direct inoculation of man. To do this experimentally is, of course, impossible, consequently we are forced to rely for evidence of this nature on those accidental cases which occur from time to time. It has been my fortune to have three such cases come under my observation, in each of which the infecting organism was known positively to be of bovine origin.[6] Similar cases have been reported by Tscherning[7] and Pfeiffer,[8] the latter ending in general infection and death. To these may be added two cases observed by Dr. M. B. Hartzell,[9] of the University of Pennsylvania, though in both

* The streptococcus is said to become increased in virulence for mice by successive passages through these animals, but less virulent for rabbits.

absolute proof of the bovine origin of the offending organism is lacking. Both occurred in healthy men employed by one of our large American railways to clean and repair cars used in the transportation of cattle. In both a well-developed tuberculosis of the skin followed slight wounds of the back of the hand inflicted by broken timbers. In one case the local disease was soon cured and no further trouble resulted. The other, however, ended fatally after about a year, though the infection becoming generalized, with involvement of the lungs. This patient was a robust man, aged forty-four years, weighing 175 pounds, with good family and personal histories. Dr. Hartzell felt able to exclude with reasonable certainty any other source of infection.

Cases such as these permit us to deny with authority the claim which has been made by certain persons that by long residence in animals of the bovine species the tubercle bacillus becomes so changed as to render it incapable of successful residence in the tissues of man. In all of these instances the bovine bacillus grew and multiplied under conditions known to be most unfavorable to it, with the production of characteristic lesions, and in two of the seven cases gained access to the internal organs, causing death, a result which is unusual when the local lesion is due to infection from human sources. While the number of cases is too small to enable us to draw sweeping conclusions, the indications are that by this mode of inoculation the pathogenic power of the bovine bacillus is at least as great as that possessed by its human congener.

*Infection through Food.* It will not be necessary here to review at length the reported instances of infection through the digestive tract following the use of food products from tuberculous cattle. Most of these, of necessity, lack precision and are not absolutely demonstrative, though some of them have, as Nocard has said, "almost the value of an experiment."

The well-known observations of Stang,[10] Demme,[10] Gosse,[10] and Ollivier[11] leave little doubt of the power of the bovine tubercle bacillus to infect man through the digestive tract.

*Statistical Evidence.* The evidence derived from the statistics of tuberculosis on this point is purely circumstantial, yet of such strength as to be most convincing. The compilations of Dr. Tatham bring out most strikingly the fact that in early life some potent factor is at work in causing tuberculosis. In the Harben Lectures for 1898, Sir Richard Thorne speaks of this as follows: "So, also, if you will compare the rates in Tables A, B, and C

(pages 5, 6, and 7), and contrast the reduction of 27.9 per cent. which has taken place, under five years of age, during the last forty-five years in all forms of tubercular disease, and that of 66 per cent. in phthisis, with the corresponding one from tabes mesenterica, which only reached 3 per cent, you will see that in considering the latter cause of death we are dealing with a totally different state of affairs.

"The matter, too, assumes a still more serious aspect if we limit ourselves to the first year of life, when milk is most largely used as food; for then we find that the reductions in the rate of death from the various forms of tuberculosis, which reduction has been going on at 'all ages' for about half a century, not only disappears, but is actually transformed into a large increase, reaching no less than 27.7 per cent. This in itself is grave enough, but its significance is still further emphasized when we remember what are the circumstances under which this increase in the rate of death from tabes mesenterica has gone on synchronously with a decrease in that from other forms of tuberculosis."

Evidence of a similar nature is given by Dr. Still,[12] of the Great Ormond Street Hospital for Children, in his analysis of 769 consecutive autopsies of children under twelve years of age, 269 of whom showed tuberculous lesions. Of these 117, or 43.5 per cent., were in children under two years of age, while in the first three years of life 56.5 per cent., or more than a half of the total number, occurred. From his study of the lesions in these cases Dr. Still believes that in 153, or 56.8 per cent., the respiratory tract was the channel of infection, while in 63, or 23.4 per cent., the alimentary canal was responsible, the remaining fifty-three cases being uncertain or were otherwise accounted for. Accepting these figures as given, they indicate strongly that milk, the most largely used food, has a considerable part in the spread of tuberculosis, and justify the conclusion quoted from a report made to the council of the British Medical Association, that "the mortality from tuberculosis in early childhood is not decreasing as it is at other ages in the United Kingdom, and the opinion that this great prevalence of the disease in childhood is due to infection through the alimentary canal by milk from tuberculous cows appears to be well founded."

From Germany comes further confirmatory facts. Widerhofer gives an analysis of 418 cases of tuberculosis in children, showing among them 101 with involvement of the intestine. Of these 43, or 42.5 per cent., were between the ages of two and five years, the period of life when cow's milk forms a large part of the food for children.

*Conclusions.* In view of the foregoing experiments, and of the evidence quoted, it seems justifiable to conclude—

1. That the tubercle bacillus from bovine sources has in culture fairly constant and persistent peculiarities of growth and morphology, by which it may tentatively be differentiated from that ordinarily found in man.

2. That cultures from the two sources differ markedly in pathogenic power, affording further means of differentiation, the bovine bacillus being very much more active than the human for all species of experimental animals tested, with the possible exception of swine, which are highly susceptible to both.

3. That tuberculous material from cattle and from man corresponds closely in comparative pathogenic power to pure cultures of the tubercle bacillus from the two sources, for all animals tested.

4. That it is a fair assumption from the evidence at hand, and in the absence of evidence to the contrary, that the bovine tubercle bacillus has a high degree of pathogenic power for man also, which is especially manifest in the early years of life.

### References.

1. Transactions of the Association of American Physicians, 1896.
2. Arkansas Agricultural Experiment Station, Bulletin No. 57, June, 1899.
3. Journal of Experimental Medicine, vol. iii., Nos. 4 and 5, 1898.
4. Annales de l'Institut Pasteur, vol. xii., No. 9, September, 1898.
5. La Revue de la Tuberculose, April, 1898.
6. Philadelphia Medical Journal, July 21, 1900.
7. Congrès pour l'étude de la Tuberculose, First Session, 1888.
8. Zeitschrift f. Hygiene, Bd. iii., 1888.
9. Journal of the American Medical Association, April 16, 1898.
10. Les Tuberculoses Animales. Nocard.
11. Paris Academy of Medicine. La Semaine Médicale, February 25, 1892.
12. British Medical Journal, August 19, 1899, and Journal of Comparatve Pathology and Therapeutics, vol. xii., Part 4.

